# Does Far Cortical Locking Improve Fracture Healing in Distal Femur Fractures: A Randomised, Controlled, Prospective Multicentre Study

**DOI:** 10.3390/jcm12247554

**Published:** 2023-12-07

**Authors:** Thomas England, Humza Khan, Sheldon Moniz, David Mitchell, Markus S. Kuster

**Affiliations:** 1 Department of Orthopaedics, Sir Charles Gairdner Hospital, Nedlands, WA 6009, Australia; tbjengland@gmail.com; 2 Department of Orthopaedics, Royal Perth Hospital, Perth, WA 6000, Australia; hk.humzakhan@gmail.com (H.K.); monizsheldon@gmail.com (S.M.); 3 Department of Orthopaedics, Ballarat Base Hospital, Ballarat, VIC 3350, Australia; david.mitchell@ballaratosm.com.au

**Keywords:** far cortical locking, bridge plate, distal femur fracture

## Abstract

(1) Background: Bone healing is influenced by various mechanical factors, such as stability, interfragmentary motion, strain rate, and direction of loading. Far cortical locking (FCL) is a novel screw design that promotes bone healing through controlled fracture motion. (2) Methods: This study compared the outcome of distal femur fractures treated with FCL or SL (standard locking) screws and an NCB plate in a randomised controlled prospective multicentre trial. The radiographic union scale (RUST) and healing time was used to quantify bone healing on follow-up imaging. (3) Results: The study included 21 patients with distal femur fractures, 7 treated with SL and 14 treated with FCL screws. The mean working length for patients with SL screws was 6.1, whereas for FCL screws, it was 3.9. The mean RUST score at 6 months post fracture was 8.0 for patients with SL plates and 7.3 for patients with FCL plates (*p* value > 0.05). The mean healing time was 6.5 months for patients with SL plates and 9.9 months for patients with FCL plates (*p* value < 0.05). (4) Conclusions: Fractures fixed with SL plates had longer working lengths and faster healing times when compared to FCL constructs, suggesting that an adequate working length is important for fracture healing regardless of screw choice.

## 1. Introduction

There are two commonly described pathways of fracture healing. The first occurs by primary bone healing with internal cortical bridging and no callus formation, while the second pathway occurs with external callus formation [1]. This is the foundation on which fracture fixation has been based since the advent of surgical fracture intervention. Mechanobiology is the study of the effect of physical stimuli on biological processes [2]. Bone healing is influenced by various mechanical factors that have been described in the mechanobiology of bone healing [2]. Epari et al.’s review primarily focused on sheep studies, they discussed a number of factors and their effects on bone healing which included stability, interfragmentary motion (IFM), strain rate, number of loading cycles, defect size, direction of loading and timing of healing [2]. 

The greater the rigidity the less external callus is formed in primary bone healing. More flexible constructs show callus formation. This, however, does not necessarily translate to higher mechanical strength. An optimum stability or fracture motion might exist to promote bridging callus and fracture healing. 

Kenwright and Goodship showed that fracture motion of 0.5 mm compared to 1 mm and 2 mm showed improved mineralisation and increased fracture stiffness in a 3 mm osteotomy gap [3]. The influence of fracture gap and amount of fracture motion was further investigated by Claes et al. [4,5]. They found that increasing the fracture gap from 2 mm to 6 mm resulted in a significant reduction in bending stiffness of the healed bone. An IFM of 30% in small gaps of 1 mm and 2 mm resulted in greater callus formation than in larger gaps (6 mm). Hence, it seems a mechanically ideal environment would have small fracture gaps of 1–2 mm with IFM of 0.3 to 0.6 mm [4,5]. 

A plate’s working length is defined as the distance between the screws on either side of the fracture and is known to affect axial stiffness and torsional stability [6]. In a biomechanical study the effects of screw placement on the stiffness of a plate bone construct were investigated [6]. This study changed the thinking from the early rigid plate osteosynthesis to a more flexible and biological plating technique [6]. Distal femur fractures remain a challenge despite increased working length and the awareness of dynamic plate osteosynthesis. Locked plating constructs have been the standard for the treatment of distal femoral fractures and have shown failure rates of 16–22% in the literature [7,8,9,10,11,12,13].

Based on the mechanobiology of fracture healing, Bottlang et al. developed the idea of far cortical locking (FCL) screws as an alternative to the standard locking (SL) screw to promote secondary bone healing [14,15,16,17,18,19]. Additionally, the direction of loading has an influence on fracture healing. It seems that axial loading is preferential to torsion or shear [20]. The FCL construct was compared to SL plate constructs in a biomechanical study [16]. It was found that initial stiffness was lower in axial compression. Almost parallel IFM at the osteotomy site was also observed in FCL constructs. Much like external fixators, FCL constructs offer a combination of fixed-angle connections between a connecting element and bone segments. In FCL, the screws extend towards the effective length of the external fixator pins. In contrast, traditional locked constructs have screws that are firmly held within the boundaries of the near and far cortices, resulting in a limited effective length that hinders flexible fixation [14,18].

This novel strategy was investigated further in an ovine study and then in a cadaveric study [17,18]. The ovine study reviewed healing fractures stabilised with FCL screws and demonstrated greater callus formation in a symmetrical distribution at the osteotomy site compared with SL. Additionally, they were found to be stronger in torsion and sustained greater energy to failure when tested after sacrifice of the animals and retrieval of the construct. The cadaveric study applied periarticular locking plate constructs to stabilise femur fractures, whereby the diaphyseal fragment was secured with either FCL or SL screws and the metaphyseal fragment was secured with standard cancellous locking screws. The FCL constructs again showed significantly less stiffness, more IFM with parallel movement of both cortices, and were comparable in terms of load to failure against an SL construct.

Despite the theoretical advantages of FCL compared to a standard bridging plate such as parallel IFM, reduced initial stiffness and controlled fracture motion are reported, and no clinical study in humans exists to prove the superiority of FCL screws compared to SL screws in a bridging plate construct with an increased working length. 

This study proposes to look specifically at callus formation and fracture healing time in older, more osteoporotic patients treated with FCL versus standard locking plate fixation, with the inclusion of periprosthetic distal femoral fractures. Fixation in these fractures is generally more challenging, and therefore it is of interest to establish whether FCL is a valuable option for these cases. Fracture healing by secondary healing with callus formation was the principal measurable outcome to demonstrate the potential differences in fixation between FCL and SL screws.

## 2. Materials and Methods

### 2.1. Design, Randomisation, and Recruitment

#### 2.1.1. Study Design and Inclusion/Exclusion Criteria

**Figure 1:**
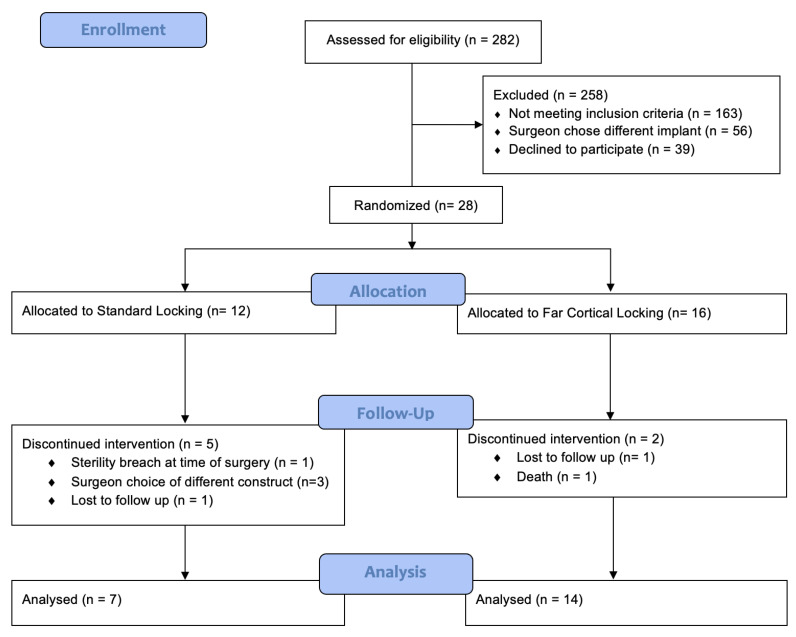
Consort Flow Diagram: Over the 3-year study period, 282 distal femur fractures were deemed suitable for plate osteosynthesis. A total of 258 patients were excluded prior to randomisation. A total of 12 were allocated to SL treatment group and 16 to the FCL treatment group. In the SL group, 7 were analysed after 5 patients had their intervention discontinued. In the FCL group, 14 were analysed after 2 had their intervention discontinued.

We conducted a randomised controlled prospective multicentre trial assessing bone healing through the use of a radiographic union scale (RUST score) to quantify bone healing on follow-up imaging. Elderly patients above the age of 60 years with acute distal femur fractures (AO type 33A and 33C) as well as hip and knee periprosthetic fractures (Vancouver type C and Rorabeck type 1 and 2) appropriate for distal femur locking plate treatment were included in the study. Young patients below the age of 60, open fracture patterns, pre-morbid non-ambulating patients and pathological fractures were excluded in our study. Trauma surgeons were given the option to fix fractures in accordance with the constructs assessed in this study; however, some chose not to use the non-contact bridging (NCB) plate assessed in this study, which slowed down the recruiting process. The recruitment period was from 1 January 2017 to 31 December 2019. The recruitment process, randomisation, and follow-up are outlined in Figure 1. 

#### 2.1.2. Randomisation

Simple randomisation was performed electronically through an online system based in Sydney, New South Wales, whereby the treating team called a 24 h randomisation hotline. Using this system, patients were assigned to an SL or FCL treatment group. 

#### 2.1.3. Recruitment

The trial was conducted with four participating sites. To ascertain a statistical power of 80% and an alpha value of 0.05, we determined that a minimum of 35 patients in each arm of the study would be required, with a discernible difference of 2 points in the RUST score at 3 months post-surgery. To accommodate for potential dropouts, our recruitment target was set at 100 patients. Notably, distal femoral fractures in elderly patients are infrequent injuries, and when such cases arose the on-call surgeon unfortunately often favoured a different implant, which made the recruiting process frustratingly slow. Regrettably, due to the unprecedented challenges posed by the COVID-19 pandemic the trial was stopped with 21 patients recruited for the analysis.

### 2.2. Procedures, Fracture Healing, and Demographic Information

#### 2.2.1. Procedures

All patients followed a routine surgical care pathway comprising pre-operative care, surgical treatment, post-operative care and follow-up outpatient review. The surgical treatment was divided into either the FCL or SL group. The fractures were treated with a lateral subvastus approach in both groups using an NCB Distal Femur Plate (Zimmer-Biomet, Warsaw, IN, USA) over the fracture site in each case and standard NCB locking screws at the distal end. The working length of each construct was aimed to be around 4 to 6 holes. The intervention group had 3–4 FCL screws (MotionLoc screw NCB, Zimmer- Biomet) used at the proximal plate end, while the control group had 3 to 4 standard NCB locking screws (NCB screw, Zimmer-Biomet). Bridged plating was applied in both groups. Routine post-operative care involved weightbearing as tolerated. Follow-up visits for clinical and radiological evaluation occurred at 6 weeks, 12 weeks, 18 weeks, 26 weeks (±14 days), and 1 year. The patients had an X-ray at each visit and CT imaging at the three-month visit to monitor the progress of healing.

#### 2.2.2. Baseline Demographic Information

Demographic information was obtained at the time of first admission (typically by the admitting staff in the emergency department). All complications (local or general) were recorded as secondary outcomes. Included complications were screw or plate failure. Other secondary outcomes included delayed union or non-union based on CT scans or X-rays and the above primary RUST scores.

### 2.3. Outcomes

#### 2.3.1. Primary Outcome

Radiologic fracture healing was the primary outcome and assessed with the RUST score using X-ray and CT scans. Two researchers (HK and MSK) assessed the progress of fracture healing for each patient. The radiographic union scale (RUST score) was used to quantify the progress of fracture healing at 3 months, 6 months and 1 year (Kooistra et al. 2010) [21]. A minimum score of 4 points represents no callus at all, while a maximum of 12 points represents healed with all four cortices bridged with callus formation and no visible fracture line. If a solid bridging callus was visible on at least two cortices, the fracture was considered healed for the calculation of the healing time.

#### 2.3.2. Secondary Outcome

All complications (local or general) were recorded as secondary outcomes. Included complications were screw or plate failure. Additionally, the rate of callus formation was assessed within the spectrum of the primary outcome as a time factor element of a successful outcome.

### 2.4. Data Management and Analysis

#### 2.4.1. Data Management

A paper record of patient data entered by a site nurse at time of initial presentation was kept. Data was sent to the central hub and entered electronically on a database. Data analysis was performed using Microsoft Excel, and statistical significance between groups (SL vs. FCL) was determined using a two-tailed unequal variance *t*-test. *P* values greater less than 0.05 were considered statistically significant. Radiographs were captured on a compact disc (CD) or the Picture Archive and Communication System for analysis by two orthopaedic surgeons (HK and MSK). 

Statistical advice was obtained from the Department of Medical Physics at Royal Perth Hospital.

#### 2.4.2. Analysis Plan

Participants were reviewed clinically and radiologically at 6 weeks, 12 weeks, 18 weeks, 26 weeks and 1 year. Clinical analysis involved patient history and examination with data recorded in a questionnaire. Radiological analysis was conducted via plain film radiographs (X-rays) and a single computed tomography (CT) scan at the three-month follow-up. Data analysis took place at the completion of the trial and involved the following methods of assessment:

- RUST score analysis;
- Plate and screw failure/breakage at six months;
- CT callus qualification of bridging at three months;
- Healing time.

## 3. Results

A total of 21 patients (7 with SL plates; 14 with FCL plates) were included in our analysis (Table 1). A total of 19 (90.5%) were female and 2 (9.5%) were male with a mean age of 87 years. Seven patients presented with a native distal femur fracture (AO classes: 33A2.1, 33A2.1, 33A2.2, 33C2.1/2, 33A3.3, 33A3.1/.2, 33A3.1/.2). Three of these underwent fixation with an SL plate and four received an FCL plate. Fourteen patients had periprosthetic fractures (4× Vancouver C and 10× Rorabeck type 2), four underwent fixation with an SL plate and ten received an FCL plate. 

**Figure 2:**
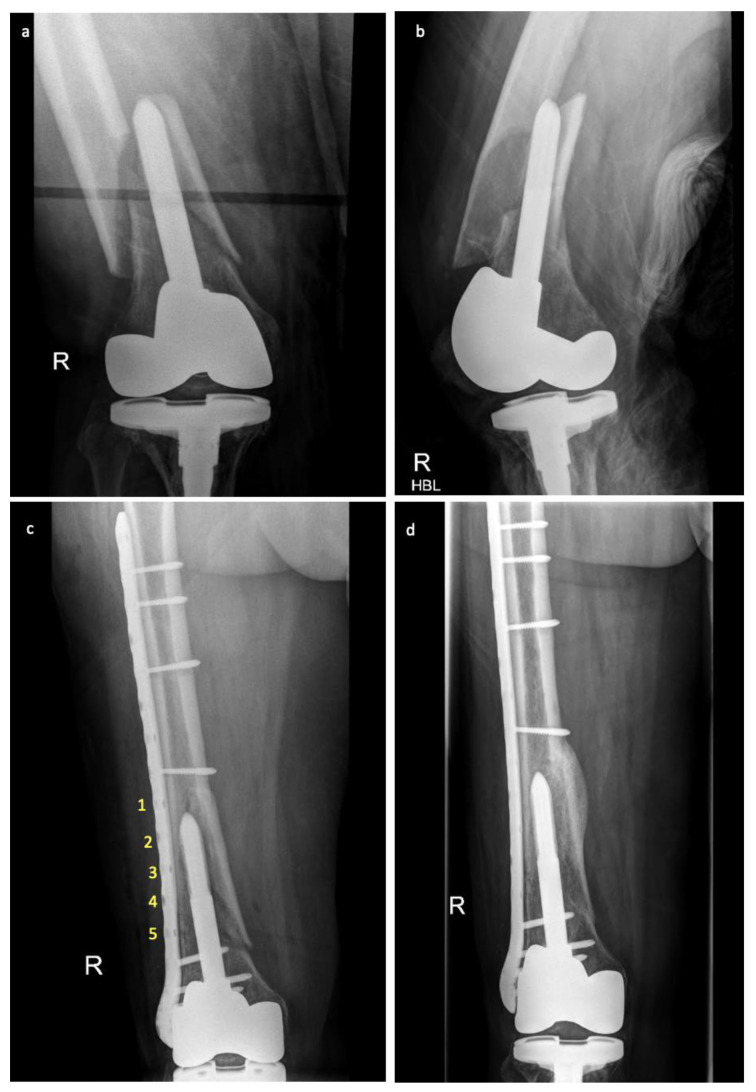
Plain film radiographs pre- and post-fracture fixation with far cortical locking screws for a periprosthetic distal femur fracture. (**a**) Anterior-posterior and (**b**) lateral views of the initial fracture. (**c**) Anterior-posterior view (working length labelled 1–5) day one and (**d**) six months post-fracture fixation with far cortical locking screws and plate constructs showing evidence of callus formation.

The average working length for the 21 patients treated with either SL or FCL fixation was 4.7 holes. Patients with SL plates had an average working length of 6.1 (range 5–9), and patients with FCL plates had an average working length of 3.9 (range 2–6; *p* value < 0.01) (see Figure 2).

The mean RUST scores in patients with an SL plate were 6.4 (range 5–10) and 8.0 (range 5–10) at 3 and 6 months post-fracture fixation, respectively. In patients with an FCL plate, RUST scores were 6.8 (range 6–10) and 7.3 (range 4–10), at 3 and 6 months post-fracture fixation, respectively. Our results reflect a mean overall RUST score of 6.6 at 3 months (*p* value > 0.05) and 7.5 at 6 months (*p* value > 0.05) following surgery. 

In patients with an SL plate, the mean healing time was 6.5 months (range 6–8 months), whereas in patients with an FCL plate the mean healing time was 9.9 months (range 5–12 months). The mean time for all patients was found to be 8.8 months (*p* value < 0.05). A post hoc power calculation (using G*Power 3.1.9.6) with our observed results gave a power of over 92% for healing time as a primary parameter.

## 4. Complications

In our series, we recorded three postoperative complications, all in elderly female patients. The first was an 86-year-old-female, on bisphosphonate therapy, who sustained a subtrochanteric fracture at the proximal end of her SL plate 6 months after fixation of her distal femur periarticular fracture. She received an intramedullary nail, leaving the SL plate in situ. Her periarticular distal femur fracture was healed at 8 months post-plate insertion. The second case involved an 88-year-old female who sustained a periarticular distal femur fracture and was initially treated with an FCL plate. However, at 6 months post operation, minimal callus formation was observed, prompting revision to a distal femur replacement. Finally, the third case involved an 85-year-old female who underwent revision to an intramedullary nail for SL plate fracture at 1 month post fixation of a native distal femur fracture. Plain film radiographs show healing at 12 months. 

## 5. Discussion

By design, the mechanical stiffness of SL plates is greater than that of FCL plates. The more rigid the construct, the more IFM is restricted. Therefore, in mechanically stiff SL plates there is a higher risk of non-union as motion necessary for the formation of callus is limited [22,23]. By design, the FCL screws have a shaft with thread on the end-segment and a non-threaded near-segment, allowing purchase of the far cortex and micromotion of the near cortex [23,24]. The entire construct is more tolerant to loads, allowing for parallel movement of the near–far cortical envelope [23]. Increased IFM permitted by FCL constructs promotes callus formation and fracture healing in comparison to conventional SL screw fixation.

In our cohort, the mean time it took for fractures to heal was 8.8 months; however, there was a statistically significant difference in the healing times between fractures managed with SL plate fixation and those managed with FCL plating. SL constructs were associated with faster fracture healing times with a mean time of 6.5 months, whilst patients with an FCL construct had a slower time to fracture healing as this was achieved at a mean of 9.9 months (*p* value < 0.05). Therefore, we were unable to establish a statistically significant improvement in fracture healing time associated with the FCL plate, instead we found that healing times were faster with SL plates. 

Screw placement from the fracture site determines the working length and contributes to the mechanical stiffness of the plate. Screws placed close to the fracture create a stiffer construct with a lower capacity for interfragmentary motion (IFM). As such, most surgeons will prefer long plate constructs for diaphyseal/metaphyseal femur fracture fixation to distribute the strain over an increased working length. In doing so, the strain at the site of the fracture is reduced [5,6]. In our analysis, we found that there was a significant difference between the working length of fractures fixed with SL and FCL plates. Fractures managed with SL fixation had longer working lengths with an average of six holes; however, those managed with FCL fixation had an average working length of four holes. Screw fixation near to the fracture site reduces the working length of the plate and creates a more rigid construct [23,24]. It is possible that the slower healing time in the FCL plate cohort was influenced by the lower working length. 

We believe that our findings are important as we unexpectedly observed a relatively slow healing process in distal femur fractures. This emphasises the need for careful consideration of the temporal aspect in fracture management. Additionally, our results highlight the critical importance of an adequate working length. Lastly, our investigation challenges the notion that dynamic FCL screws outperform SL screws. Contrary to expectations, we found SL screws not to be inferior when an adequate working length is applied. These insights will hopefully contribute valuable information to our understanding of fracture healing dynamics and have potential implications for clinical practice.

Our analysis encountered certain limitations that should be taken into consideration. Firstly, the sample size of our study was relatively small resulting in reduced statistical power of the analysis conducted. Secondly, the combination of reduced recruitment and the external randomisation process resulted in an unequal distribution of FCL plates (*n* = 14) compared to SL plates (*n* = 7), potentially introducing a bias that might have influenced the outcomes of our investigation. Thirdly, we assumed that all patients were compliant with postoperative weight bearing instructions; however, rates of touch weight bearing noncompliance have been recorded as high as 75%. This would increase the risk of implant failure and delayed fracture healing [23,24].

## 6. Conclusions

Fracture fixation and healing in distal femur fractures is a clinical challenge with a long healing time in most cases. The NCB distal femur plate is a safe and valuable option with either standard locking screws or far cortical locking screws. FCL screw fixation was developed as a means to improve fracture healing. Whilst other studies have demonstrated a theoretical mechanical benefit for FCL plate fixation over SL constructs, we have been unable to reproduce this in vivo. The present study further emphasises that an adequately long working length is important for fracture healing irrespective of screw choice.

## Figures and Tables

**Table 1:** Patient demographics and fracture fixation results. Patients’ sex, age, native, and periprosthetic fractures fixed with standard (SL) or far cortical locking (FCL). Fracture fixation results demonstrated as working length, RUST score at 6 months and average healing time in months.

	Total	SL	FCL	*p* Value
Number of patients	21	7	14	
Males n (%)	2 (9.1%)	0	2	
Females n (%)	19 (90.9%)	7	12	
Mean Age (years)	86.5	86.3	81.4	
Number of native fractures	7	3	4	
Number of periprosthetic fractures	14	4	10	
Average working length	4.7	6.1	3.9	<0.005 *
Average RUST Score at 3 months	6.6	6.4	6.8	>0.05
Average RUST Score at 6 months	7.5	8	7.3	>0.05
Average healing time (months)	8.8	6.5	9.9	<0.05 *

* Statistically significant result.

## Data Availability

The data supporting the findings of this study are available on request from the corresponding author. The data is not publicly available due to restrictions that could compromise the privacy of research participants.
